# Development of Chitosan Nanoparticles as a Stable Drug Delivery System for Protein/siRNA

**DOI:** 10.1155/2013/146320

**Published:** 2013-09-30

**Authors:** Haliza Katas, Maria Abdul Ghafoor Raja, Kai Leong Lam

**Affiliations:** Centre for Drug Delivery Research, Faculty of Pharmacy, Universiti Kebangsaan Malaysia, Jalan Raja Muda Abdul Aziz, 50300 Kuala Lumpur, Malaysia

## Abstract

Chitosan nanoparticles (CS NPs) exhibit good physicochemical properties as drug delivery systems. The aim of this study is to determine the modulation of preparative parameters on the physical characteristics and colloidal stability of CS NPs. CS NPs were fabricated by ionic interaction with dextran sulphate (DS) prior to determination of their storage stability. The smallest CS NPs of 353 ± 23 nm with a surface charge of +56.2 ± 1.5 mV were produced when CS and DS were mixed at pH 4 and with a DS : CS mass ratio of 0.5 : 1. An entrapment efficiency of 98% was achieved when BSA/siRNA was loaded into the nanoparticles. The results also showed that particle size and surface charge of CS NPs were slightly changed up to 2 weeks when stored at 4°C. Greater particle size and surface charge were obtained with increasing the concentration of DS. In conclusion, NPs were sufficiently stable when kept at 4°C and able to carry and protect protein.

## 1. Introduction

Endogenous peptides, protein, and oligonucleotides are among the main drugs which attract much attention because of their great potentials in treating chronic diseases [1]. However, the extreme *in vivo* environment of human body has always limited the therapeutic applications of these substances [2, 3]. Polymeric nanoparticles have attracted much attention as delivery systems due to their ability in overcoming the physiological barriers and protecting and targeting the loaded substances to specific cells [4, 5]. Naturally occurring polymers such as chitosan (CS) have been studied to form nanoparticles [6, 7]. CS is a biodegradable polysaccharide, and it is derived from deacetylation of chitin [8]. Apart from its biocompatibility, the low toxicity, hemostatic, and bacteriostatic properties also contribute to its various applications in pharmaceutical field [9–11]. Several anions have been investigated to crosslink CS like sodium sulphate [12] and dextran sulphate (DS) [13]. DS is able to modify protein and siRNA entrapment efficiency (EE) without the use of hardening agents and control the rate of drug release due to its high charge density [14]. Besides DS is a cheap material [15], it produces mechanically more stable nanoparticles compared to the pentasodium tripolyphosphate (TPP) [16, 17].

Several studies had reported the unique features of chitosan nanoparticles (CS NPs) using DS. However, the modulation of preparative parameters on their physical characteristics is still not fully investigated, for example, the influence of DS steric hindrance on the electrostatic attraction between CS and BSA [18]. Furthermore, the determinant of a successful drug delivery system is dependent on its physical characteristics and stability. Therefore, the objectives of present study were to modulate preparative parameters to obtain nanosized particles of CS NPs and to determine their colloidal stability at different storage temperatures and in various suspending mediums.

## 2. Materials and Methods

### 2.1. Materials

Low molecular weight chitosan (70 kDa with the degree of deacetylation 75%–85%), acetic acid glacial, phosphate buffered saline (PBS), bovine serum albumin (BSA, 46 kDa), and Bradford reagent was purchased from Sigma-Aldrich Inc., USA. Double-stranded siRNA (sense: 5′-GAUUAUGUCCGGUUAUGUAUU-3′, antisense: 3′-UACAUAACCGGACAUAAUCUU-5′) was purchased from Thermoscientific Dharmacon, USA. Dextran sulphate (DS) was purchased from Fisher Scientific, UK. Protein ladder (High range), Laemmli sample buffer, 10x tris/glycine/sodium dodecyl sulfate buffer, ammonium persulfate, tetramethylenediamine (TEMED), 2% bis solution, and 40% acrylamide solution were purchased from Bio-Rad, USA. Tris-HCl buffer was obtained from Invitrogen, USA. All other chemicals used were of analytical grade.

### 2.2. Preparation of Blank and BSA-Loaded CS NPs

CS and DS solution were dissolved in 1% v/v acetic acid and distilled water, respectively. pH of CS solution was adjusted to pH 4 by adding 1 M NaOH or 1 M HCl. DS solution (0.05%, 0.1%, 0.15%, 0.2%, and 0.25% w/v) was added dropwise into CS solution (0.1% w/v) under magnetic stirring (WiseStir Digital Multipoint Magnetic Stirrer MS-MP8, DAIHAN Scientific, Korea) at 250 rpm for 15 min to form nanoparticles. All samples were made in triplicate. The resultant nanoparticles were washed and harvested by ultracentrifugation (Optima L-100 XP Ultracentrifuge with a rotor NV 70.1, Beckman-Coulter, USA) twice at 12 500 rpm for 15 min at 10°C. For BSA association into CS NPs, BSA was dissolved in CS solution (0.1% w/v, pH 4) to produce a final concentration of 1 mg/mL. BSA-loaded CS NPs were then prepared by the above method. For siRNA association into CS NPs, 3 *μ*L of siRNA (15 *μ*g/*μ*L) in deionized water was added to DS solution (0.05%, 0.1%, 0.15%, 0.2%, and 0.25% w/v) before adding this dropwise to CS solution (0.1% w/v).

### 2.3. Electrophoretic Mobility Study

Electrophoretic mobility measurements (*μ*_e_) of CS NPs were performed with a Zetasizer Nano ZS (Malvern Instruments, UK) and *μ*_e_ was measured against waiting time. Each sample was analyzed in triplicate.

### 2.4. Nanoparticles Characterization

Particle size, surface charge, and polydispersity index (PDI) of freshly prepared CS NPs were measured using a Zetasizer Nano ZS (Malvern Instruments, UK), based on the Photon Correlation Spectroscopy (PCS) techniques. No dilutions were performed during the analysis. Each sample was analyzed in triplicate. The measurements were made at 25°C.

### 2.5. Morphological Analysis

Morphological characterization of unloaded CS NPs, BSA/siRNA loaded CS NPs (DS : CS weight ratio of 0.5 : 1, 1 : 1) was carried out by using transmission electron microscopy (TEM), Tecnai Spirit, FEI, Eindhoven (The Netherlands).

### 2.6. BSA/siRNA Entrapment Efficiency

BSA/siRNA loaded CS NPs were separated from the solution by ultracentrifugation (Optima L-100 XP Ultracentrifuge with a rotor NV 70.1, Beckman-Coulter, USA) at 14000 rpm for 30 min. Supernatants recovered from centrifugation were decanted. BSA content in the supernatant was analyzed by a UV-Vis spectrophotometer at 595 nm (U.V-1601; Shimadzu, Japan) using the Bradford protein assay as per manufacturer instruction. siRNA content in the supernatant was analyzed by a UV-Vis spectrophotometer at 260 nm. Samples were prepared and measured in triplicate. The BSA/siRNA entrapment efficiency (EE) was calculated using the following equation:

(1) EE(%)=(Total  amount  of BSA/siRNA added)−(Free  amount  of BSA/siRNA)(Total  amount  of BSA/siRNA added) ×100.

### 2.7. Stability of CS NPs

Freshly prepared CS NPs (made from 0.05% and 0.1% w/v of DS and CS solution, resp.) were centrifuged at 12 500 rpm for 15 min prior to storing. After ultracentrifugation, the obtained pellets were resuspended in either distilled water (measured pH of 6.6) or PBS pH 7.4. The particle size and surface charge were measured at predetermined storage time durations (0, 1, 2, 3, 5, 8, and 14 days), and at either ambient temperature or 4°C.

### 2.8. *In Vitro* Drug Release Study

The release of BSA/siRNA was determined from CS NPs with the highest EE (DS : CS ratio 1 : 1, EE = 98% ± 0.2 and 95 ± 4, resp.). BSA/siRNA loaded CS NPs were suspended in Tris-HCl buffer solution (pH 7.4, 4 mL) and placed on a magnetic stirrer with a stirring speed of 100 rpm at 37°C (MS MP8 Wise Stir Wertheim, Germany) for 48 h at 37°C. At predetermined time intervals (0, 0.5, 1, 2, 4, 6, 12, 20, 24, and 48 h), samples were centrifuged at 14 000 rpm for 30 min at 10°C. Then, the supernatant was decanted and replaced with an equivalent volume of fresh buffer solution. The amount of released BSA/siRNA in the supernatant was analyzed by a UV-Vis spectrophotometer (U.V-1601; Shimadzu, Japan) at a wavelength of 280 and 260 nm, respectively.

### 2.9. BSA Integrity

The integrity of BSA released from CS NPs was determined by SDS-PAGE (12% resolving and 10% stacking gel) using Mini-Protein System (Bio-Rad, USA). BSA samples were mixed with Laemmli sample buffer in 1 : 1 ratio and heated at 95°C for 5 min. Samples (15 *μ*L) were loaded into the wells and the gel was run using a Mini-Protein System Tetra Cell at a constant voltage of 150 V for 90 min with a running buffer containing 25 mM Tris, 192 mM glycine, and 0.1% SDS at pH 8.3. The sample bands were stained for 40 min with 0.1% Coomassie blue solution containing 40% acetic acid and 10% methanol, followed by staining overnight with a solution of 40% acetic acid and 10% methanol.

### 2.10. Statistical Analysis

All the data were presented as mean ± standard deviation (SD). Statistical analysis (ANOVA test and Tukey's posthoc analysis) was performed by using the Statistical Package for the Social (SPSS) programme version 15. A *P* value < 0.05 showed significant difference between the mean of tested groups.

## 3. Results

### 3.1. Particle Size and Surface Charge

**Figure 1:**
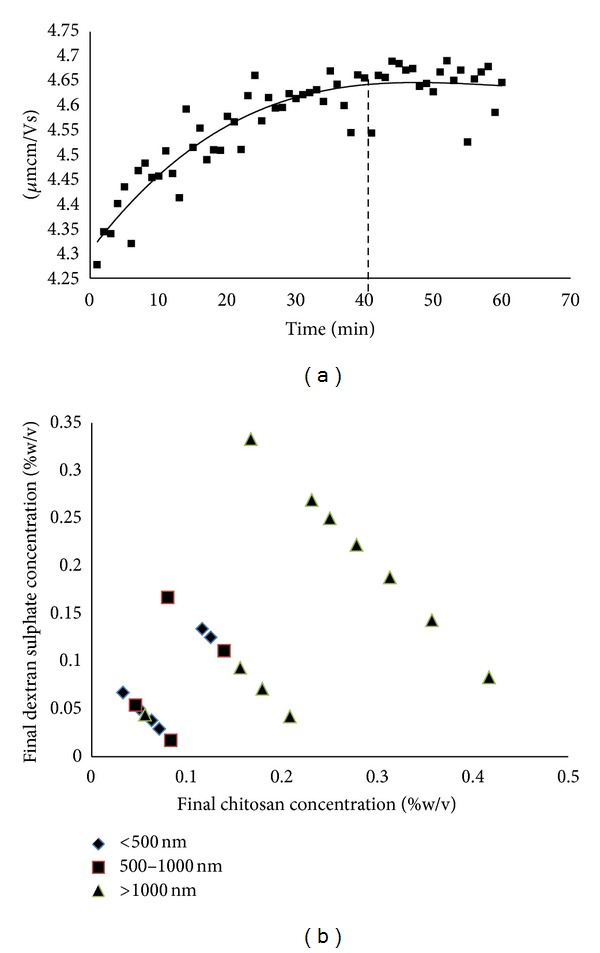
Electrophoretic mobility as a function of time (a) and effect of the final concentrations of CS and DS on the particle size of nanoparticles (b), *n* = 3.

Figure 1(a) demonstrates the results of electrical mobility (*μ*_e_) against waiting time. From the graph, it could be observed that the *μ*_e_ remained plateau and constant after 30 min. This demonstrates that the formation of stable electrical double layer (e.d.l.) was not instantaneous but required some moments. The effects between CS concentration and DS final concentrations on the size of CS NPs are presented in Figure 1(b). It was observed that most of the CS NPs with the size of less than 500 nm were obtained at a low CS concentration (0.1% w/v). DS concentration also influenced the size of nanoparticles (*P* < 0.05). An increasing trend in particle size could be observed with increasing the DS concentration from 0.05 to 0.25% w/v. In general, DS concentration of 0.05% w/v (low concentration) produced nanoparticles with particle size less than 500 nm. Contrary to that, large nanoparticles (>1000 nm) were obtained when concentration of both polymers was increased to 0.25% or above. Based on the results, DS concentrations from 0.05 to 0.20% w/v were selected for the following studies. Furthermore, an increase in the DS : CS weight ratio (higher density of negative charges from DS present in the system) led to an increase in particle size but a decrease in particle surface charge (Table 1 (above)). As the CS weight exceeded the mass of DS, a positive value of +56.2 ± 1.5 mV was obtained. However, particle surface charge decreased to −34.7 ± 4.34 mV when more negatively charged DS was added. It was continuously decreasing when the DS : CS weight ratio had reached to 2.5 : 1. This was expected to be due to an excess of DS molecules accumulated on the surface of nanoparticles.

Table 1 (below) shows that DS 0.2% w/v possessed the largest particle size after being loaded with BSA. The particle size was 1127 ± 247 nm. Particle size for DS at concentration of 0.1 and 0.15% w/v was also larger than the empty ones (*P* < 0.05). On the other hand, higher positive values of surface charge were observed for BSA loaded nanoparticles compared to the empty ones. This was observed for all DS concentrations. Moreover, higher EE values could be achieved by increasing the DS : CS weight ratio above 0.5 : 1. The EE of nanoparticles at DS : CS weight ratio of 1 : 1, 1.5 : 1, and 2 : 1 was 98.6 ± 0.2%, 88.5 ± 5.0%, and 91.5 ± 3.1%, respectively. The highest EE was obtained at a DS : CS weight ratio 1 : 1 (Table 1 (below)).

Table 2 shows that DS 0.2% w/v possessed the largest particle size (900 ± 60 nm) after being loaded with siRNA. siRNA loaded CS NPs at different DS concentrations (0.05, 0.1, 0.15, and 0.2% w/v) showed smaller particle size. The EE of nanoparticles at DS : CS weight ratio of 0.5 : 1, 1 : 1, 1.5 : 1, and 2 : 1 was 94 ± 3%, 95 ± 4%, 92 ± 2%, and 90 ± 2%, respectively.

### 3.2. Morphology

**Figure 2:**
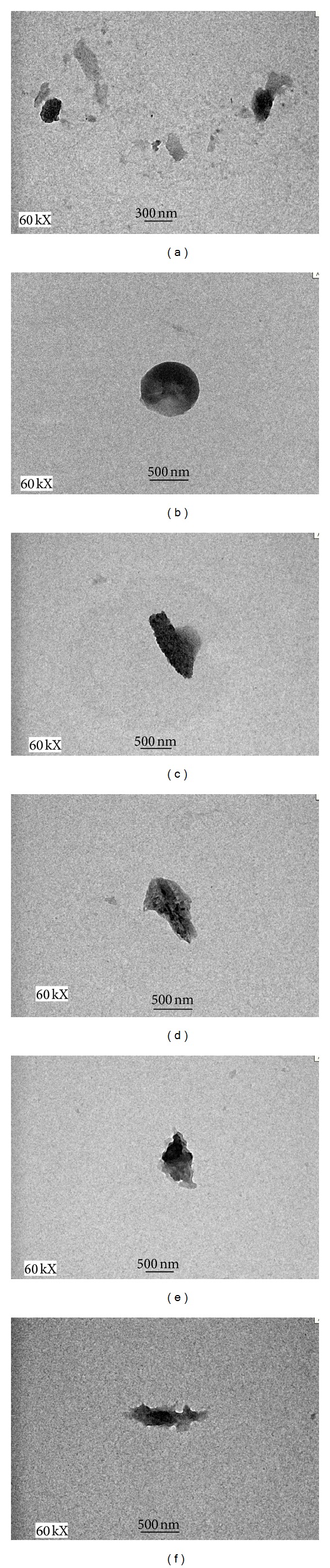
TEM images of CS NPs. (a) and (b) Unloaded CS NPs at 0.5 : 1 and 1 : 1, (c) and (d) BSA loaded CS NPs at 0.5 : 1 and 1 : 1, and (e) and (f) siRNA loaded CS NPs at 0.5 : 1 and 1 : 1, respectively. All the images were taken at 60 kX magnification.

The images of the CS NPs were obtained by TEM (Figure 2). Figures 2(a) and 2(b) show that unloaded CS NPs exhibited a spherical structure. The images demonstrated that nanoparticles generated from siRNA (Figures 2(e) and 2(f)) showed irregular morphology; however, BSA loaded nanoparticles displayed elongated morphologies (Figures 2(c) and 2(d)).

### 3.3. Storage Stability of CS NPs

**Figure 3:**
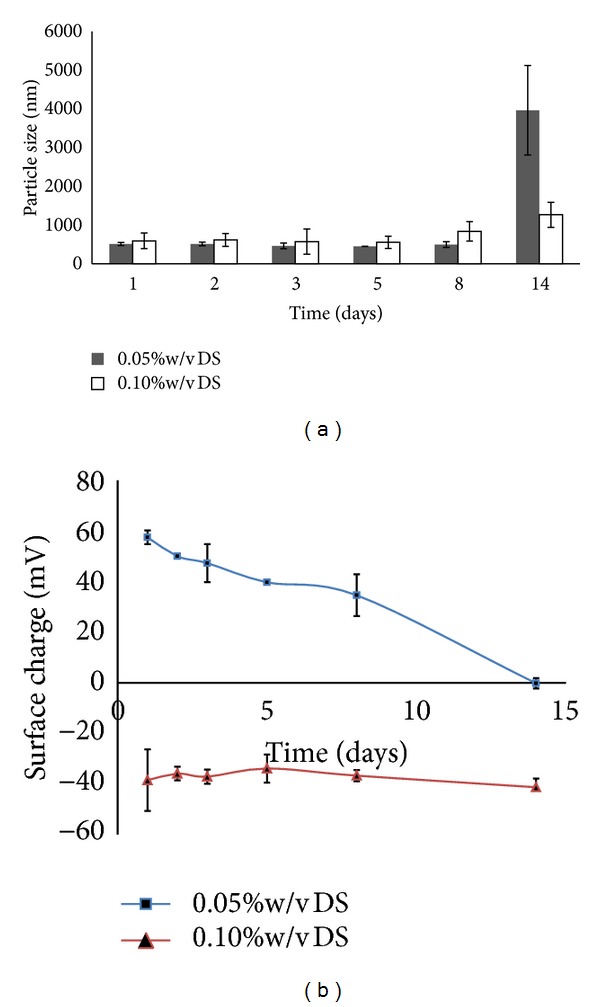
(a) Particle size and (b) surface charge of CS NPs prepared at 0.05 and 0.01% w/v DS solution and stored at 25°C. Nanoparticles were suspended in distilled water (pH in the range of 6-7), *n* = 3.

**Figure 4:**
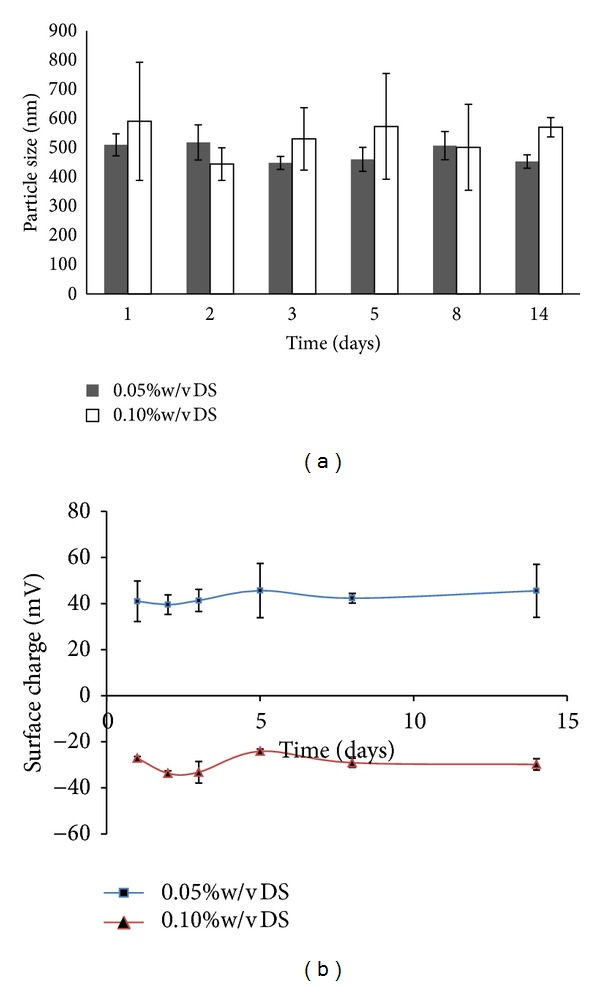
(a) Particle size and (b) surface charge of CS NPs prepared at 0.05 and 0.10% w/v and stored at 4°C. Nanoparticles were suspended in distilled water (pH in the range of 6-7), *n* = 3.

Both nanoparticles made from 0.05 and 0.10% w/v DS were increased in size over time as shown in Figure 3(a) when stored at ambient temperature. A significant increase in particle size was observed after day 14 of storage especially for 0.05% w/v DS. This was thought to be due to the formation of aggregates. This finding corroborated with the results of surface charge which showed a decrease in surface charge to nearly neutral. In contrast, when they were stored at 4°C, their particle size and surface charge remained unchanged up to 14 days for nanoparticles made from 0.10% w/v DS. A slight change was observed for 0.05% w/v DS (Figures 4(a) and 4(b)). On the other hand, when these nanoparticles were suspended in PBS pH 7.4, all formulations were aggregated to larger sizes of more than 1 *μ*m with PDI values more than 0.5. Their particle surface charges were also nearly neutral, ranging from +0.2 to +2.5 mV.

### 3.4. BSA *In Vitro* Release and Integrity

**Figure 5:**
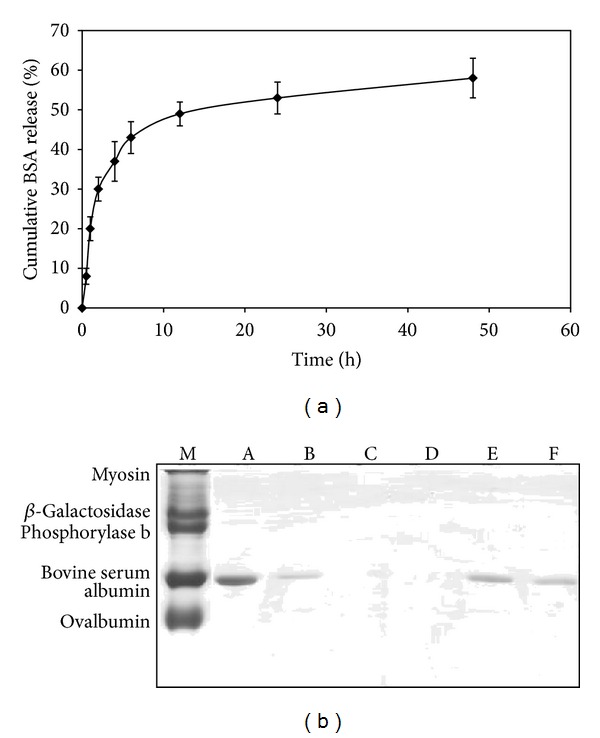
(a) The release profile of BSA loaded CS NPs at DS : CS ratio of 1 : 1 at pH 7.4, *n* = 3. (b) SDS-PAGE analysis of BSA released from CS NPs: (M) SDS-PAGE standards (BIO-RAD); (A) BSA standard 1 mg/mL; (B) BSA standard 0.2 mg/mL; (C) blank; (D) unloaded CS NPs; (E) and (F) BSA released from CS NPs (DS : CS ratio of 1 : 1) at days 1 and 2.

Figure 5(a) illustrates that the release of BSA could be divided into two stages based on the release rate. In the first stage, the BSA was rapidly released from the CS NPs and showed a burst release in the first 6 h. This resulted in a 45% ± 5 cumulative release of BSA. In the second stage, BSA was slowly released from 6 h up to 48 h, resulting in a cumulative BSA release of more than 60%. The integrity of BSA released from CS NPS was evaluated by SDS-PAGE and is shown by Figure 5(b). The bands observed confirmed that BSA that had endured the loading and release processes at 37°C after days 1 and 2 were not different from that of freshly prepared BSA standards. Therefore, it could be concluded that BSA remained in its native form in the CS NPs under the experimental conditions.

### 3.5. siRNA *In Vitro* Release

**Figure 6:**
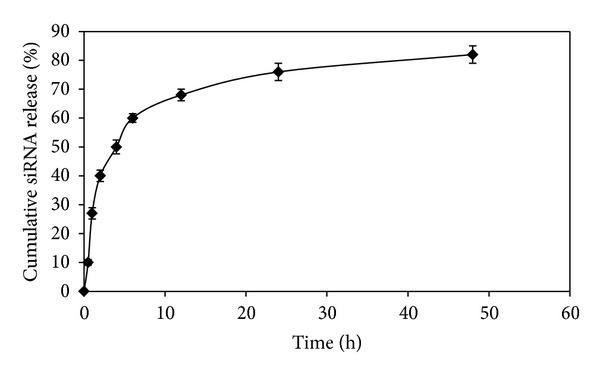
The release profile of siRNA loaded CS NPs at DS : CS ratio of 1 : 1 at pH 7.4, *n* = 3.

Figure 6 illustrates that the release of siRNA could be divided into two stages based on the release rate. In the first stage, the siRNA was rapidly released from the CS NPs and showed a burst release in the first 6 h. This resulted in a 58% ± 5 cumulative release of siRNA. In the second stage, siRNA was slowly released from 6 h up to 48 h, resulting in a cumulative BSA release of more than 85%.

## 4. Discussions

The method used to produce CS NPs in the present study is a mild process, and it enables control of the particle size by varying certain parameters for example, concentration of added salts, viscosity, quantity of nonsolvent, and molecular weight of polymer. This study was started with the investigation to obtain information regarding electrical state of ionizable groups of CS NPs by determining the stabilization time of e.d.l. This step is important to obtain reliable and reproducible *μ*_e_ results. The data obtained suggested that formation of stable e.d.l. during nanoparticles preparation required some moments after stopping the stirring. These moments were needed in order for the electrolytes to penetrate towards the particles nucleus. Thus, waiting time of 40 min was needed before *μ*_e_ of CS NPs could be accurately measured. This finding was similar to the CS-tripolyphosphate (CS-TPP) NPs which suggested the same waiting time [16].

A study was also carried out to determine the influence of polymer concentration on particle formation. The study was aimed at establishing the range of polyelectrolytes concentration to produce nanoparticles with the desired size. To study the effects of the varying concentrations of CS and DS on the formation of nanoparticles, CS and DS solution of 0.1, 0.25, and 0.5% w/v were prepared. Variable volumes of DS solution (1, 2, 3, 4, 5, 5.8, and 10 mL) were mixed with 5 mL of each CS concentration (0.1–0.5% w/v). The final concentration of CS and DS was calculated, and sizes of samples were categorized either as 100–500, 501–1000, or more than 1000 nm. It was found that particle size was affected by the DS concentration. This finding corroborated with the results of CS-TPP NPs [19]. In general, the desired size of nanoparticles confined between 100 and 1000 nm. However, previous studies [19, 20] have shown that the loaded nanoparticles would normally produce a larger size than the empty ones. The size of below 500 nm is therefore favorable.

Furthermore, the results revealed that only DS concentration of 0.05% w/v was able to produce nanoparticles with particle size less than 500 nm as shown in Table 1. It was expected as when both polymers were in low concentrations, the addition of DS to the CS resulted in small coacervate nuclei. Contrary to that, large coacervates which exceeded 1000 nm in size tended to form when both polymers concentration increased to 0.25% or above. Chitosan's ability of spontaneously forming coacervate is due to the interaction of oppositely charged polyelectrolytes to form a polyelectrolyte complex with reduced solubility. The mixture of high concentration of DS with CS is therefore more likely to affect the entanglement of the CS chains and solubility of the resulted complex. As a result, a high degree of complexation and coacervate will be produced [21]. The decreased viscosity at a lower concentration of CS also resulted in better solubility. This allowed for a more efficient interaction between the cationic CS and oppositely charged ions, and thus a smaller particle size was produced [22]. In addition, an increase and excess in the molar mass of the polyanion used resulted in larger particles because highly neutralized complexes were formed and they tended to flocculate [15]. In this study, particle surface charge of the nanoparticulate system was dependent on the weight ratio of DS and CS. Particle surface charge was found to be increased as the ratio decreased. This relationship could be useful in obtaining the desired particle surface charge density to facilitate adhesion and transport properties of the nanoparticles.

In present study, the incorporation of BSA into CS NPs was achieved by simply mixing the acidic CS solution containing dissolved BSA molecules with the DS solution at room temperature without addition of stabilizer. BSA is frequently used as a model protein because it embraces the general characteristic of other proteins and it is biocompatible to humans. It was found that CS NPs were comparatively larger in size after loading with BSA. Particle size was expected to increase when BSA was successfully being loaded into nanoparticles. This trend may be possibly due to the molecular weight and size of the added BSA molecules. These large particle sizes may limit their use in delivery of protein. Nanoparticles of 150–300 nm are found mainly in the liver and the spleen [23]. Besides, according to some reports, the “ideal” size requirement for nanoparticles developed for cancer treatment is between 70 and 200 nm [24]. Although nanoparticles should be not greater than 150 nm to cross the endothelial barrier, the literature always reports the penetration of particles larger than the limits of fenestrations. Indeed, fenestration and the vasculature can undergo modification under various pathological conditions [25].

For instance, tumor growth will induce the development of neovasculature characterized by discontinuous endothelium with large fenestrations of 200–780 nm [26]. Besides, it was observed that the particle surface charge of BSA loaded nanoparticle was higher than the empty ones. This may be due to the cationic characteristic possessed by BSA when present in acidic condition. The positive charges from CS and BSA molecules therefore have contributed to a higher value of particle surface charge for the loaded nanoparticles.

Positively charged cationic polymers can effectively bind to and protect nucleic acids such as DNA, oligonucleotides [27], and siRNA [22]. In this study, the incorporation of siRNA into CS NPs was achieved by simply mixing the acidic CS solution with the DS solution containing siRNA at room temperature. It was found that particle size of CS NPs was comparatively smaller in size after loading with siRNA. The smaller size of CS NPs loaded with siRNA could be due to neutralization of negative charges of nucleic acid by cationic polymer resulting in condensed smaller sized nanoparticles. The siRNA loaded CS NPs also showed higher zeta potential than blank CS NPs, following the same trend as that of BSA loaded CS NPs.

Ideally, a successful delivery system should have a high degree of associating drugs. The siRNA loaded CS NPs showed higher entrapment efficiency (<90%) for all DS : CS weight ratios. The entrapment efficiency of nanoparticles at DS : CS weight ratio of 1 : 1, 1.5 : 1, and 2 : 1 was higher than the weight ratio of 0.5 : 1. This phenomenon was most probably due to higher proportion of DS presented in the nanoparticles. As more DS added, it would facilitate more BSA to be entrapped into nanoparticles. This could be explained by the fact that BSA is a zwitterionic molecule. At the pH of formulation medium of 3.5–4.0, the solubility of BSA could be highly increased due to increased positive charges possessed by it [21]. Thus, BSA would be able to electrostatically attach and stably load into the nanoparticles. In acidic solution, BSA could possess positive charge and compete with CS to interact with DS electrostatically. This finding was corroborated with the increased positive surface charges of BSA loaded CS NPS compared to unloaded ones. Moreover, there are multi-ionic sites on BSA, and this feature could facilitate the incorporation of BSA into nanoparticles. This finding differs from the finding with CS-TPP NPs [19].

In the study, the electrostatic interaction was present between BSA and CS, instead of BSA and TPP. It was also suggested that BSA should dissolve in a solution with pH higher than its isoelectric point in order for BSA to possess negative charge and interact with the positively charged CS molecules. This finding therefore demonstrated that electrostatic interaction is the main contributing factor to promote the incorporation of BSA into nanoparticles either via CS-protein interaction or DS-protein interaction.

TEM allows nanoscale visualization of individual nanoparticles and provides information of both size and morphology. The particle morphology is an important factor for the colloidal and chemical stability as well as the bioactivity of nanoparticles. siRNA loaded CS NPs showed irregular morphology; however, BSA loaded CS NPs showed elongated morphology. This could be due to larger size of BSA which may entangle or act like a shield to CS, thus limiting the overall exposure of CS within structure.

Stability profile of CS NPs upon storage is also important. This information could provide a view about the stability of nanoparticles under different media and temperature. The stability of nanoparticles was investigated by assessing their variation in mean particle size and surface charge over time. At first, the nanoparticles were resuspended in distilled water at pH 6.6 which was filtered by 0.2 *μ*m filter to remove possible contaminants present in water. For this study, only nanoparticles made from 0.05 and 0.10% w/v of DS were tested. Other DS concentrations were not determined due to increased particle size after centrifugation. The particle size was up to 1643 ± 442 nm and 2218 ± 587 nm for 0.15% and 0.20% w/v DS, respectively. The increment of particle size may due to CS NPs themselves erode and lose their spherical shape in an aqueous environment, and consequently the mean diameter of particle would rise as response to this erosion [16]. Furthermore, particle surface charges for the nanoparticles made from both concentrations were decreasing over time. It was suspected that CS may be degraded in aqueous media even though in absence of lysozymes. The results showed that CS NPs were more stable when stored at 4°C as their particle size and surface charge were unchanged or slightly changed up to 14 days. The results also suggested that CS NPs should not be stored at ambient temperature as they are prone to degradation. The results therefore suggested that CS NPs stored at room temperature are more prone to degradation than those that were stored in cool environment. It was probably due to the cool environment which may slow down the kinetic motion of nanoparticles. Thus, the nanoparticles could maintain their spherical shape and erosion would be less likely to occur. Moreover, it was observed that these nanoparticles were aggregated in PBS at pH 7.4. This may attribute to lower particle surface charge of nanoparticles in PBS, near to neutral. Particle surface charge which dropped to zero may indicate that CS NPs had undergone charge cancellation by phosphate groups of PBS. The neutral charged status of these nanoparticles may cause losing of intra- and intermolecular forces, important to maintain the nanoparticles individually. As a result, these uncharged nanoparticles may start to aggregate and destabilize the colloidal system. In contrast to PBS, distilled water can provide numerous hydrogen ions to form hydrogen bonds which can assist in breaking aggregation of nanoparticles by interacting with ionizable groups of CS NPs.

The *in vitro* release study of BSA and siRNA from CS NPs was carried out in Tris-HCL buffer. The release of BSA and siRNA could be divided into two stages based on the release rate. In the first stage, the drug was rapidly released from CS NPs. The release of BSA and siRNA at this stage might involve the diffusion of BSA/siRNA bound at the particle surface. In the second stage, BSA/siRNA was released slowly due to swelling or degradation of the polymer. The remaining BSA/siRNA in CS NPs would not completely be released until the particles were completely eroded or dissolved in release medium. This might have been due to the interaction between the remaining BSA/siRNA and free amino group on the CS segments [28, 29]. In addition, the synthesized system which has been previously described as being able to be formulated under mild conditions assured that the stability of the proteins loaded into CS NPs was intact as determined by SDS-PAGE.

## 5. Conclusions

In summary, this study shows that CS and DS concentration as well as pH were the parameters controlling particle size and surface charge of CS NPs. Nanoparticles less than 500 nm could be obtained at DS : CS weight ratio of 0.5 : 1 at pH 4. In the case of BSA entrapment, nanoparticles with higher DS : CS weight ratios have possessed higher entrapment efficiencies of more than 88%. The highest percentage of entrapment efficiency achieved was at 0.10% w/v DS (DS : CS ratio of 1 : 1). However, CS NPs loaded with siRNA showed high entrapment efficiency (>90%) for all DS : CS ratios. Storage temperature and suspending medium were found to be the factors that could influence the stability of CS NPs. CS NPs were labile and tend to destabilize at ambient temperature but withhold this labile behavior when cool environment (2–4°C) was provided. In addition, CS NPs had better stability in distilled water than in PBS which might be due to hydrogen bonds that formed between water molecules and ionizable groups of CS NPs.

## Figures and Tables

**Table:** (a)

DS (% w/v)	CS (% w/v)	DS : CS weight ratio	pH of nanoparticle dispersion	Particle size, nm ± SD	PDI ± SD	Surface charge, mV ± SD
0.05	0.10	0.5 : 1	3.84	353 ± 23*	0.34 ± 0.06*	+56.2 ± 1.5*
0.10	0.10	1 : 1	3.79	701 ± 72	0.59 ± 0.11	−34.7 ± 4.34*
0.15	0.10	1.5 : 1	3.80	809 ± 133	0.68 ± 0.17	−45.1 ± 2.6*
0.20	0.10	2 : 1	3.81	877 ± 132	0.68 ± 0.12	−50.5 ± 2.43*
0.25	0.10	2.5 : 1	3.82	1083 ± 311*	0.77 ± 0.16	−54.7 ± 3.27*

**Table:** (b)

DS (% w/v)	CS (% w/v)	DS : CS weight ratio	Particle size, nm ± SD	PDI ± SD	Surface charge, mV ± SD	EE, % ± SD
0.05	0.10	0.5 : 1	526 ± 14*	0.45 ± 0.02	+63.8 ± 5.8*	50.5 ± 2.6*
0.10	0.10	1 : 1	833 ± 66	0.42 ± 0.06	+17.9 ± 0.2*	98.6 ± 0.2*
0.15	0.10	1.5 : 1	958 ± 66	0.45 ± 0.07	−26 ± 2.3*	88.46 ± 5.0
0.20	0.10	2 : 1	1127 ± 249*	0.45 ± 0.06	−38.4 ± 3.9*	91.5 ± 3.1

*The mean difference is significant at the 0.05 level.

**Table 2:** Effects of DS : CS weight ratios on the physical characteristics of siRNA loaded CS NPs, *n* = 3. CS solution used for BSA loaded CS NPs was 0.1% w/v. siRNA concentration used was (15 *µ*g/*µ*L).

DS (% w/v)	CS (% w/v)	DS : CS weight ratio	Particle size, nm ± SD	PDI ± SD	Surface charge, mV ± SD	EE, % ± SD
0.05	0.10	0.5 : 1	330 ± 50*	0.45 ± 0.01	63.6 ± 5.9	94 ± 3
0.10	0.10	1 : 1	540 ± 30*	0.50 ± 0.02	53.0 ± 4.0	95 ± 4
0.15	0.10	1.5 : 1	776 ± 40*	0.51 ± 0.01	20.1 ± 5.2*	92 ± 2
0.20	0.10	2 : 1	900 ± 60*	0.60 ± 0.04	6.0 ± 4.0*	90 ± 2

*The mean difference is significant at the 0.05 level.
